# Pathogenesis of prostate cancer and hormone refractory prostate cancer

**DOI:** 10.4103/0970-1591.30265

**Published:** 2007

**Authors:** J. S. Girling, H. C. Whitaker, I. G. Mills, D. E. Neal

**Affiliations:** CRUK Uro-oncolgy Group, Department of Oncology, University of Cambridge, Hutchison/MRC Research Centre, Cambridge, CB2 2XZ, UK

**Keywords:** Prostate cancer, refractory androgen receptor

## Abstract

Prostate cancer is the second most common malignancy in males and the leading cause of cancer death. Prostate cancer is initially androgen dependent and relies upon the androgen receptor (AR) to mediate the effects of androgens. The AR is also the target for therapy using antiandrogens and LHRH analogues. However, all cancers eventually become androgen independent, often referred to as hormone refractory prostate cancer. The processes involved in this transformation are yet to be fully understood but research in this area has discovered numerous potential mechanisms including AR amplification, over-expression or mutation and alterations in the AR signaling pathway. This review of the recent literature examines the current knowledge and developments in the understanding of the molecular biology of prostate cancer and hormone refractory prostate cancer, summarizing the well characterized pathways involved as well as introducing new concepts that may offer future solutions to this difficult problem.

Prostate cancer is the commonest malignancy in men, accounting for 24% of all cancers in the UK in 2003.[1] The treatment of prostate cancer varies with the disease stage at diagnosis and includes surgical, radiotherapeutic and medical interventions. Initially, prostate cancer requires androgens such as testosterone, or the more potent dihydrotestosterone (DHT), for growth and is therefore referred to as androgen dependent.[2] Steroidal androgens exert their effects by binding to the androgen receptor (AR) in the cytoplasm of cells promoting nuclear translocation. Once in the nucleus the AR binds to specific DNA sequences androgen response elements (ARE's) and promotes transcription of androgen-regulated genes that control cellular growth, differentiation and apoptosis. One such gene is prostate specific antigen (PSA) the well characterized marker of prostate cancer.

As a result, treatment for locally advanced and metastatic prostate cancer targets the AR by reducing the levels of androgens or by inhibiting the activation of the AR. Androgens are removed by either surgical or chemical methods using luteinising-hormone-releasing-hormone (LHRH) analogues with or without the addition of antiandrogens. Initially, prostate tumors regress in response to androgen deprivation in up to 80% of cases.[3] However, tumors eventually begin to grow, despite continued antiandrogen treatment, progressing to metastatic and ultimately fatal prostate cancer within 24-48 months.[4] Tumor growth in these late stages is termed androgen independent or hormone refractory. The mechanism of progression from androgen dependent to independent disease remains poorly understood.[5–7] It is thought that tumor cells either bypass or alter the AR activation pathway to allow continued growth, e.g., by AR amplification, mutation or modification or that hormone refractory cells may express aberrant levels of cofactors or activate the AR via alternative signaling pathways. This review examines the current knowledge of the molecular biology of prostate cancer and hormone-resistant prostate cancer focusing on the putative pathways involved in the progression to hormone refractory disease.

## THE ANDROGEN RECEPTOR (AR)

**Figure 1:**
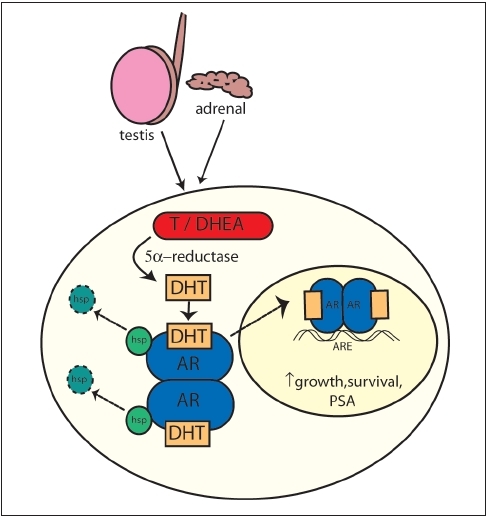
Intracellular metabolism of androgens. AR - androgen receptor, T - Testosterone, DHEA - Dehydroepiandrosterone, DHT-Dihydrotestosterone, hsp - heat shock protein, ARE - Androgen response elements

**Figure 2:**
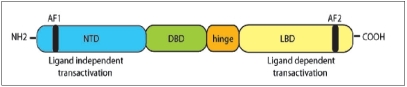
Schematic representation of androgen receptor showing the NTD - N-terminal domain, DBD - DNA-binding domain, LBD - ligandbinding domain, AF - activation function domains 1 in N-terminus and 2 in LBD

Androgens are essential to the normal development and biology of the prostate. The majority of testosterone is produced by the testes, with a smaller contribution of androgens (5%) from the adrenal glands. These androgens are metabolized by 5α-reductase to dihydrotestosterone (DHT) which binds to the androgen receptor (AR). Ligand binding initiates phosphorylation, homodimerisation of the AR and dissociation of heat-shock proteins, allowing translocation of the AR complex to the nucleus. Here the AR binds to specific DNA sequences called androgen response elements (ARE) promoting transcription of androgen-responsive genes. Such genes control a range of cellular events such as growth, differentiation and apoptosis [Figure 1]. The AR is a member of the steroid receptor superfamily and functions as a ligand-dependent transcription factor. The majority of the nuclear receptors, including the AR, share a common structure composed of four domains: an N-terminal domain (NTD), central DNA-binding domain (DBD), hinge region and C-terminal ligand-binding domain (LBD) [Figure 2]. Transcription is mediated by two activation function domains (AF-1 and AF2). The AF2 is contained within the LBD and the binding of the hormone induces the conformational changes necessary for its activation. However, unlike other steroid receptors the AF-1 region of the AR has been shown to be most important for transactivation and can be activated even in the absence of hormone.[8] This is supported by recent data demonstrating that activation of AF-1 alone is responsible for AR activation in hormone refractory cells, even in the presence of antiandrogens.[9] This would clearly be possible if the ligand binding does not effect conformational changes in the NTD.

As hormone refractory prostate cancer continues to grow in the presence of antiandrogens the term hormone independence may be misleading as it implies that the AR is no longer required for growth. However, the AR is expressed throughout the progression of prostate cancer, including >80% of hormone refractory tumors, suggesting that AR signaling remains essential.[10,11] It has also been shown that over-expression of the AR alone is sufficient to promote hormone refractory disease.[12] Indeed, the persistence of AR in the metastatic LNCaP cell line and continued nuclear expression of hormone refractory tumor samples is indicative of continued AR involvement.[13]

Amplification of the AR gene is rarely identified in untreated cases of prostate cancer. Using comparative genome hybridization (CGH) and fluorescent in-situ hybridization (FISH), 30% of treated cases exhibited AR amplification.[11,14,15] Patients exhibiting amplification have been shown to have an improved response to hormone manipulation with a relapse time of >12 months.[14] Identical techniques were also used to identify chromosomal aberrations, among which 8q amplification was the most consistent finding in up to 90% of locally advanced and metastatic tumors.[16] Amplification of the AR gene does not always result in over-expression of AR mRNA or protein.[16] However, several studies have shown a correlation between AR amplification and AR protein expression.[17,18] Conversely, AR over-expression does occur in the absence of AR amplification[11] and there is growing evidence that in all except the small minority of small cell prostate tumors AR is ubiquitously expressed. In particular, AR expression is known to be increased in hormone refractory disease allowing activation by adrenal androgens. Using micro-array genome-wide profiling of xenograft models comparing androgen ablation treated and untreated prostate cancer, Chen *et al* demonstrated that over-expression of AR cDNA, mRNA and protein levels were the only consistent and significant differences.[12] In patients, AR over-expression conferred a better response to combined androgen blockade but despite this it also correlates with poorer outcome.[19]

**Figure 3:**
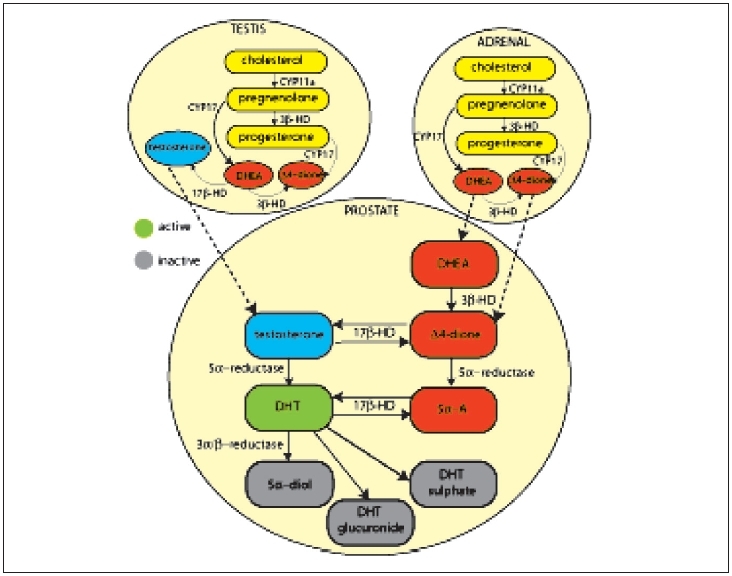
Schematic representation of adrenal, testicular and prostatic intracrine androgen metabolism. CYP17 - steroid 17-alpha-hydroxylase, CYP11a - steroid 11 - alpha - hydroxylase, DHEA dehydroepiandrosterone, 5a - A-5-androstane-3, 17 dione, DHT - dihydrotestosterone, 5a-diol - 5a androstane-3, 17 diol

Despite medical or surgical castration, tumor levels of androgens in human prostate samples, in particular DHT, have been shown to remain at levels sufficient to transactivate the AR in cell line studies.[20] Although serum testosterone is reduced by 95% following castration, tissue levels of DHT remain as high as 40%[21] suggesting that through intracrine processing within the prostate, adrenal androgens are transformed into the 10 times more potent DHT[20] [Figure 3]. Following studies in androgen-regulated cell lines which indicated an increase in apoptosis and reduction in proliferation, the use of 5α-reductase inhibitors are currently being assessed as a potential adjuvant agent.[22]

Mutations in the AR are uncommon (0-4%) in untreated prostate cancer and those treated with surgical castration. However, in hormone refractory tumors the incidence of mutations increases by up to 50%.[12] Over 70 AR mutations have been found in association with prostate cancer with the majority being point mutations resulting in a single amino acid substitution.[24] Within the AR, mutations predominantly occur in the ligand-binding domain including T887A which is also found in LNCaP, a prostate cancer cell line derived from lymph node metastases.[25–28] This mutation permits binding and activation of the AR by a wide variety of ligands including antiandrogens and estrogens, permitting cyproterone acetate and flutamide to bind and activate the AR and altering the nuclear translocation of bicalutamide-bound AR.[29] When T877A is found in association with another mutation L701H, glucocorticoids can bind and activate the receptor.[30] The H874Y mutated AR has also been shown to bind flutamide and the adrenal androgen dehydroepiandrosterone and become activated.[31] These gain-of-function mutations of AR lead to the failure of conventional antiandrogen therapies.[32] There is growing evidence suggesting that antiandrogens can select for mutated receptors capable of activation. Taplin demonstrated that only 6% of patients treated with monotherapy exhibited AR mutated compared to 31% in patients on a combined regime containing flutamide.[27] A similar study with bicalutamide saw 36% of patients with mutated ARs.[33] In both studies the mutated receptors were capable of being activated by the antiandrogens, suggesting that cells containing mutated receptors may be selected by antiandrogen treatment.

Modulation of AR activity by co-repressors and co-activators The ARE-bound AR binds a number of proteins to enable efficient recruitment of the basal transcriptional machinery and transcription of androgen-regulated genes. The transcriptional activity of the AR is enhanced by co-activators and inhibited by co-repressors although some proteins can either promote or repress transcription depending upon the cellular context.[34] Co-activators are essential for effective steroid receptor-mediated transcription. After binding to the liganded AR in the nucleus, co-activators catalyze the recruitment of chromatin-remodeling proteins such as histone acetyl-transferases (HATs). The HAT proteins modify histone tails giving rise to a more open chromatin structure capable of binding the transcription initiation machinery.[35] Recruitment of co-activators is regulated by AR acetylation by proteins such as p300 and Tip60, which is itself upregulated by androgen ablation.[36] It has been suggested that over-expression of co-activators may provide a mechanism for transition to hormone refractory prostate cancer by amplifying the response to adrenal androgens. ARA70 was first identified as an AR-specific co-activator although it has since been shown to co-activate other steroid receptors.[37,38] Probably the most extensively studied group of AR co-activators is the p160 family which includes steroid receptor co-activator -1 (SRC1), glucocorticoid receptor-interacting protein -1(GRIP-1) also known as transcriptional intermediary factor-2 (TIF-2) and receptor-associated co-activator-3 (RAC-3).

The p160 co-activators are known to interact with CREB-binding protein (CBP) and its homologue p300 which have intrinsic HAT activity.[39,40] The primary role of CBP/p300 when bound to p160 co-activators is to act as a co-integrator, collating multiple proteins into an integrated HAT response at promoters.[41] Immunohistochemical studies of CBP expression of in-tumor samples were inconclusive with Debes *et al*[42] showing an increase in hormone refractory disease whereas Linja *et al* found no significant difference in expression.[43] As yet no studies have looked at matched samples pre and postandrogen ablation therapy. The CBP has been shown to enhance the agonistic properties of flutamide, increasing AR transactivation in the prostate cancer cell lines LNCaP and DU145.[44]

Probably the best characterized co-activator of the AR is SRC-1. In cell line studies reduction of SRC-1 expression significantly reduced growth and altered AR target gene regulation in the LNCaP cell line whereas it had no effect on the growth of the AR-negative PC-3 and DU145 prostate cancer cell lines, further emphasizing the need for an intact AR.[45] In a study of cell lines and clinical prostate samples SRC-1 was expressed at higher levels in high-grade prostate cancer samples and this was supported by significant over-expression of both SRC-1 and TIF-2 in hormone refractory compared to hormone-naïve prostate cancer.[45,46] However, Linja *et al* found a decrease in the median expression of SRC-1 suggesting that sample collection, processing and the sensitivity of the detection method may influence co-activator expression studies.[43]

GRIP-1/TIF-2 has previously been shown to bind the steroid receptors including the AR via its LBD and promote transcription of androgen-regulated reporter genes. The AR mutation N727K results in sub-fertility at least in part by abrogating GRIP-1/TIF-2 binding. Using domain constructs it was shown that GRIP-1/TIF-2 enhances AF-2 but not AF-1 mediated AR transactivation.[47] Bicalutamide has been shown to block recruitment of GRIP-1/TIF-2, presumably by preventing the formation of a co-activator binding surface.[48] Using reverse transcriptase PCR GRIP-1/TIF and RAC-3 were found at low levels in prostate cancer specimens[49] suggesting it may not be as significant as SRC-1 in prostate cancer progression.

Expression of RAC-3 (also known as SRC-3) was higher in prostate cancer cell lines expressing the AR and has been shown to promote ligand-independent activation of the Akt pathway. RAC-3 mRNA and protein expression in prostate cancer cells has been shown to correlate with tumor grade and stage and increased expression correlates with poor survival in clinical studies.[50]

Adaptor proteins associated with receptor internalization at the cell surface have also been reported to be prostate cancer biomarkers and to act as transcriptional co-regulators. Two examples of these proteins are Huntingtin Interacting Protein 1 (HIP1) and cyclin G-associated kinase (GAK), both of which were initially implicated in clathrin-mediated receptor trafficking.[51,52] Subsequently, they were shown to be over-expressed in prostate cancer and in the case of GAK to be upregulated following prolonged androgen ablation therapy (greater than six months).[53,54] It was then proposed that changes in their expression could prevent the cell from effectively internalizing activated growth factor receptors for degradation and so potentiate signaling.[55,56] Both GAK and HIP1 have however been reported to associate with the AR and to co-activate the receptor.[54,57] The degree to which adaptors directly affect steroid hormone receptor signaling, as opposed to indirectly through the perturbation of growth factor receptor trafficking and signaling in prostate cancer, remains to be resolved.

Steroid receptor activity can also be modified by co-repressors which inhibit transcription by blocking co-activators binding and recruiting histone deacetylases (HDACs) and repressor proteins such as Sin3A.[58] This HDAC complex gives rise to a more condensed chromatin structure, hindering the recruitment of the basal transcription machinery. There is also evidence of co-repressor recruitment by antagonist-bound receptors suggesting that a loss of co-repressor expression could lead to increased AR transactivation. Silencing mediator for retinoid and thyroid hormone receptors (SMRT) and nuclear receptor co-repressor (N-CoR) have been identified as co-repressors of AR. The SMRT interacts with both liganded and unliganded forms of AR reducing transactivation. Recruitment is enhanced in the presence of cyproterone acetate and bicalutamide.[59,60] However, as yet there is no evidence that expression differs with prostate cancer progression. Recently, HDAC-7 has been shown to repress AR transactivation in the presence of DHT. Both cyproterone acetate and bicalutamide-bound AR co-localized with HDAC-7 in nuclear bodies suggesting it may be essential for inhibiting AR function.[61]

## LIGAND-INDEPENDENT ANDROGEN RECEPTOR ACTIVATION

**Figure 4:**
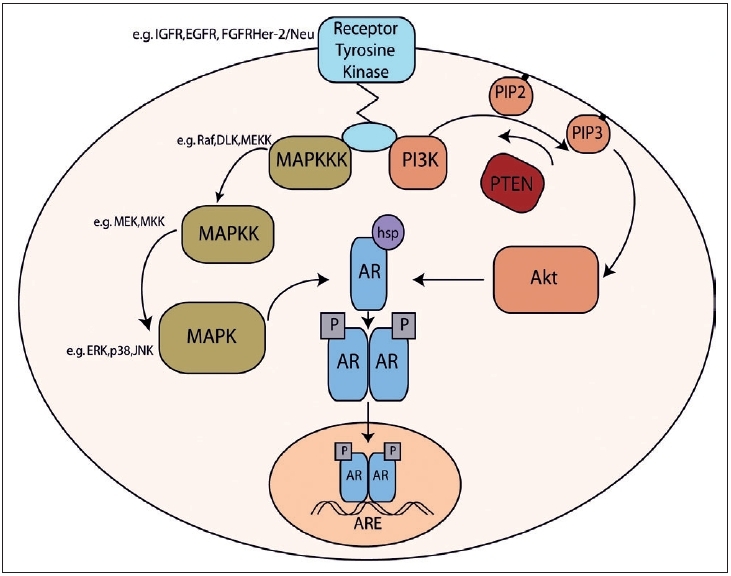
Schematic representation of the interactions between androgen receptor, MAPK and Akt signalling. IGFR - insulin-like growth factor receptor, EGFR - epidermal growth factor receptor, FGFR - fibroblast growth factor receptor, MAPK mitogen-activated protein kinases, MAPKK - MAPK kinases, MAPKKK - MAPKK kinases, DLK - Dual leucine zipper bearing kinase, MEK - Mitogen-activated protein kinase/ERK kinase, ERK - extracellular signal-related kinase, JNK - c-Jun-N-terminal kinases, PI3K- phosphoinositol 3-kinase, PIP - phosphatidylinositol phosphate, AR - androgen receptor, P - phosphorylation, ARE - androgen response elements

Classically, activation of the AR via the binding of androgens initiates receptor phosphorylation and/or acetylation, homodimerization dissociation of heat-shock proteins, allowing translocation of the AR complex to the nucleus. The AF-1 region of the N-terminal of the AR can be activated even in the absence of ligand thereby circumventing current hormonal therapies. Ligand-independent activation induces phosphorylation and acetylation of the AR altering its conformational and subsequent transactivation. Phosphorylation increases AR stability and stabilizes AR homodimers increasing AR signaling without ligand.[62] However, phosphorylation needs to occur at specific sites to permit the conformation change necessary for nuclear translocation. Many pathways have been identified as being involved in this process with the best characterized being mitogen-activated protein kinase (MAPK) and phosphoinositol 3-kinase (PI3K)-Akt [Figure 4]. Both pathways can be initiated by growth hormones such as insulin-like-growth factor (IGF), epidermal growth factor (EGF) and Her-2/Neu – an EGF-related molecule.

The PI3K-Akt pathway is involved in processes controlling cell growth, survival, cycle regulation and apoptosis. Following growth receptor activation, PI3K phosphorylates phosphatidylinositol (3, 4)-bisphosphate (PIP2) to phosphatidylinositol (3, 4, 5)-trisphosphate (PIP3). Akt and its activating kinases, phosphoinositide-dependent kinases 1 and 2 (PDK1 and PDK2), then localize to the membrane. Here, activated Akt can activate a myriad of substrates and consequently a vast number of intracellular events including phosphorylation of AR.[63] A recent study investigating the expression of the three isoforms of Akt demonstrated differential staining during prostate cancer progression. Akt1 correlated with high prostate specific antigen (PSA) whereas Akt3 correlated with invasion, metastases and hormone refractory disease.[64] PTEN is a phosphatase that opposes the function to PI3K, dephosphorylating PIP3 and deactivating Akt, thus operating as a tumor suppressor. Loss of PTEN results in Akt activation and a subsequent reduction in apoptosis. Loss of PTEN has been shown to correlate with tumor recurrence and progression to androgen independence.[65] In a model using PTEN ± mice, usually susceptible to the development of multiple tumors, the inhibition of Akt reduced the onset of malignancy, most significantly in prostate cancer reinforcing the role of Akt in prostate carcinogenesis.[66]

Mitogen-activated protein kinases (MAPK) are a family of serine/threonine protein kinases involved in many cellular programs such as cell proliferation, cell differentiation and apoptosis. The MAPK signaling cascades are organized hierarchically into three-tiered modules: MAPK-kinase kinases (MAPKKKs) are in activated by interaction with the family of small GTPases and/or other protein kinases. MAPKKKs are phosphorylated and activate (MAPKKs), which in turn phosphorylate and activate MAPK-kinases (MAPKs). The MAPK pathways terminate with the proteins JNK, ERK and p38 which are known to be involved in both anti- and pro-apoptotic processes. There is conflicting evidence regarding the significance of the MAPK pathway in prostate cancer progression. Initial reports suggested a significant correlation between the activation of MAPK enzymes ERK1 and ERK2 and tumor grade, with 70% of high-grade tumors exhibiting activation.[67] In two patients with matched pre and postablation therapy they demonstrated absence and presence of activation respectively. However, Uzgare *et al* reported that although there was a slight increase in ERK and p38 in well-differentiated cancers, cells from hormone refractory prostate cancer had reduced or absent activated expression of isoforms.[68] Furthermore, Malik *et al* found a decrease in activated form phospho-MAPK/ERK in high-grade tumors.[69]

Her-2/Neu is known to play a significant role in the progression of breast cancer raising interest in this molecule as a potential target in prostate cancer. Her2/Neu can activate AR via the MAPK and PI3K-Akt pathways and over-expression in LNCaP cells inhibits growth arrest by 35% compared to wild-type LNCaP cells. The potential importance of Her-2/Neu was highlighted in a xenograft model used to produce progressively more androgen-independent tumors, in which the level of Her-2/Neu expression increased 25-fold. This is reinforced by human immunohistochemical studies that demonstrate a significantly positive correlation between Her-2/Neu expression and hormone independent status when comparing hormone-naïve and advanced hormone refractory disease.[70,71]

Growth factors and their receptors have previously been shown to be essential for prostate growth and differentiation during development, in particular FGF-7 and 10.[62] FGF-8 and receptors 1 and 4 in particular have also been linked to prostate cancer[72] and shown to activate the MAPK and PI3K pathways. Insulin-like growth factor (IGF) is essential for the normal development and transformation of cells. IGF can activate the AR to levels similar to DHT possibly by dephosphorylation of AR serine 650 which prevents its nuclear export.[73] Activation of the AR was blocked by bicalutamide suggesting that the activation is mediated through the AR.[74] Cell studies comparing different stages of prostate cancer have also demonstrated IGF receptor (IGFR) protein expression changes. Initially IGFR is over-expressed driving malignant transformation but levels in hormone refractory prostate cancer are reduced[75] - a finding confirmed at the mRNA level in a transgenic mouse model.[76] EGF binding to the EGFR on the surface of cells is known to activate the MAPK pathway and potentiate GRIP1 co-activation of the AR.[77] Many studies have looked at the expression of EGFR (also known as ErbB1). Hernes *et al* compared biopsy samples from the same patient taken before and after the development of hormone refractory disease and found a significant increase in EGFR from 23 to 43% after hormone relapse.[78] Other studies have found no significant differences between the expression of EGFR and Her-2/Neu in matched hormone-sensitive and hormone-relapsed tumors. There was also a significant decrease in survival time following hormone treatment with increased expression of either EGFR or Her-2/Neu.[79] While the data suggests there is some variation in the significance of growth factor signaling in prostate cancer, a growing body of evidence supports the hypothesis that it is likely to be important in progression to hormone independence.

In addition to growth factor signaling other extracellular proteins are known to activate cell surface receptors and the AR. The best characterized of these proteins is interleukin-6 (IL-6), a cytokine previously linked to a number of malignancies including renal, myeloma and prostate. IL-6 has wide-ranging cellular effects consistent with activation of multiple pathways including MAPK and PI3-Akt. Cell studies have confirmed that IL-6 can induce ligand-independent AR transactivation via a mechanism which is blocked by bicalutamide and inhibitors of MAPK and protein kinase A.[80] When IL-6 expression was examined in patient serum[81] and prostate tissue[82] both demonstrated upregulation in hormone refractory cancers. IL-4 has also been shown to be upregulated in the serum of hormone refractory prostate cancer patients via activation of the AF-1 region of the AR.[83]

## THE ROLE OF PROSTATE CANCER STEM CELLS IN HORMONE REFRACTORY DISEASE

The classical stochastic model of cancer development is based on the theory that every cell within a tumor has the potential to form a new tumor colony. However, the stem cell model of tumorigenicity proposes that it is a minority of specialized stem cells that give rise to cancer cells.[84] Prostate cancer stem cells are reported to be AR-negative making them resistant to androgen ablation. The surviving stem cell is then able to proliferate resulting in a resistant tumor recurrence.[85] This would account for the heterogeneity of tumor masses whereby the stem cells then go on to develop to different degrees of differentiation and molecular characteristics and would suggest that in order to accurately characterize and treat prostate cancer, it is necessary to focus on the stem cells. Techniques have been developed to identify stem cells within tumors and primary prostate cancer cell lines by utilizing the over-expression of the integrin α_2_β_1_ and CD133 as markers.[85] However, the limited number of prostate cancer stem cells available (<0.1% of tumor bulk) has limited profiling studies to date. The potential of being able to target the tumor-initiating cells provides an exciting prospect for diagnosis and targeted therapies.

## SUMMARY

The development and progression of prostate cancer is a complex process. The androgen signaling pathway and its interaction with other pathways impacts on cellular processes from growth, cell cycle, differentiation to growth arrest and apoptosis. Through adaptation and alteration cells become tumorigenic. Initially this process can be halted by manipulating the cells’ requirement for androgens although eventually this treatment fails and cancer cells resume growth. The terms ‘androgen independent’ and ‘hormone refractory’ may be misnomers as the AR appears to maintain a role in cancer progression as demonstrated by its continued and even increased expression. There is also evidence that despite castration, both surgical and medical, the prostate retains a level of androgen that is high enough to induce AR transactivation in prostate cancer cell lines. The AR mutations and over-expression also enable transactivation to occur at low levels of androgen as well as decreasing ligand specificity. The upregulation of co-activators and possible downregulation of co-repressors further potentiate these effects. Alternative pathways involving growth factors and receptors and IL-6 have been shown to interact with the androgen signaling pathway enabling transactivation to occur even in the absence of ligand.

These processes are unlikely to operate independently. In fact the heterogeneity of prostate cancer, both hormone-naïve and hormone refractory, indicate that a multifactorial, multistep process is the most plausible. This could be a double-edged sword. The presence of numerable potential targets and markers is encouraging, particularly if tumor profiling can be utilized. However, it is also an indication of the likely complexities to be faced when designing therapies for the multiple types of prostate cancers. Recent advances have highlighted many potential and targets for pathway inhibition. By targeting multiple pathways, including AR signaling, it is feasible that it may possible to treat patients with tumor-specific ‘tailor-made’ therapies in the future.
